# Enhanced osteogenic activity of Ti alloy implants by modulating strontium configuration in their surface oxide layers

**DOI:** 10.1039/c7ra10807a

**Published:** 2018-01-15

**Authors:** Zhengjiang Xu, Huaifeng Lu, Jian Lu, Chen Lv, Xiaobing Zhao, Guocheng Wang

**Affiliations:** Research Center for Human Tissues and Organs Degeneration, Shenzhen Institutes of Advanced Technology, Chinese Academy of Science Shenzhen Guangdong 518055 China gc.wang@siat.ac.cn; School of Materials Science and Engineering, Changzhou University Changzhou 213164 China zhaoxiaobing00@163.com

## Abstract

To guarantee the long-term stability of an orthopaedic implant, non-degradable surface coatings with the ability to selectively release bioactive drugs or ions are especially desirable. In this study, SrO–TiO_2_ composite coatings were deposited on the surface of Ti alloys, whose release behavior of bioactive Sr ions was modulated by the Sr configurations, either interstitial atoms in solid solution (Ti_*y*_Sr_2−2*y*_O_2_) or strontium titanate (SrTiO_3_). A perfect linear relationship between the amount of the released Sr ions and the Sr content in the coating was observed. Among the SrO-doped TiO_2_ coatings, the 20% SrO–TiO_2_ coating where Sr existed in both forms of Ti_*y*_Sr_2−2*y*_O_2_ and SrTiO_3_ not only promoted proliferation of bone cells but also enhanced their osteogenic differentiation, which was proved to be related to its Sr release behavior. However, overdosing with 30% SrO only resulted in one single Sr configuration (SrTiO_3_) and an inferior osteogenic function. This study suggests that Sr configurations of both interstitial atoms of the solid solution and SrTiO_3_ can realize the selective release of Sr, but they possibly have different effects on the biological functions and other properties including corrosion resistance.

## Abstract 2

Strontium configurations can modulate its release in the SrO–TiO_2_ coating system, thus being able to control the interfacial osteogenesis.

## Graphical abstract

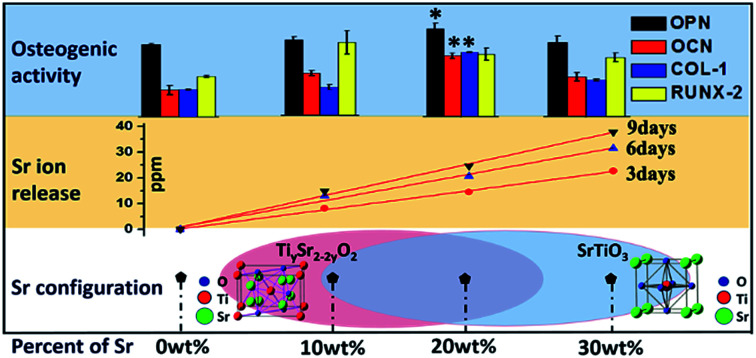

## 1. Introduction

A variety of metal ions have been documented to be essential for the human body and widely used as therapeutic agents in the treatment of many types of diseases, such as anemia, bone, brain and neuron diseases.^1–3^ In bone tissue engineering, some metal ions, represented by strontium (Sr),^4^ zinc (Zn),^5^ magnesium (Mg)^6^*etc.* were proven to promote bone tissue regeneration and have been widely used in the field of therapeutic tissue engineering. One of the most important advantages of using metal ions is that it does not pose risks of decomposition or instability, which are intrinsic to some drugs and proteins.^3,7^ Among those ions reported to be essential for bone metabolism, Sr has been demonstrated to stimulate bone regeneration and inhibit bone resorption,^8–10^ which has raised great interest in construction of osteoinductive bone biomaterials.

Titanium and its alloy are the most commonly used implant materials for dental and orthopaedic applications due to their good mechanical properties, biocompatibility and corrosion resistance.^11,12^ However, they cannot achieve sufficient functional integration (osseointegration) with the surrounding bone to establish a firm and long-lasting anchor, as the implant surface lacks osteoinductivity. Therefore, surface activation is required to obtain a satisfactory long-term performance.^13,14^ Benefiting from the osteogenic function of Sr ions, they have been used by various methods to activate Ti alloy implant surface.^15–18^ The commonest way is to introduce Sr in a biodegradable material that can be deposited onto the implant surface *via* a certain of surface techniques.^19–21^ Upon degradation of the material, the Sr ions release and the resultant biological effects on bone cells can be realized.^16^ However, since the bone implant is supposed to permanently stay in human body, the utilization of a biodegradable coating on its surface could risk the interfacial stability and probably cause implant aseptic loosening upon the coating absorption, if its degradation rate cannot match the new bone formation rate.^22^ Therefore, to better use of the osteogenic benefits of Sr and avoid the risk of implant loosening caused by the coating degradation, a chemically stable surface coating with an ability to release Sr ions would be preferred.

In this study, we choose biocompatible and chemically stable TiO_2_ as the main component of the surface coating material, which is supposed to permanently exist during the service time of the implant. Sr ions were incorporated into the TiO_2_ promote the osseointegration of the implant. To modulate the ion release behavior, the amount of Sr in the TiO_2_ was tailored in order to control the Sr configuration (Sr–TiO_2_) solid solution and strontium titanate (SrTiO_3_) in the composite coating. The release of Sr is not associated with the degradation of the whole coating material, thus avoiding the risk of compromising the implant stability resulted from by the coating degradation. The ion release behavior, corrosion resistance and bioactivity of the Sr-doped coatings were evaluated and discussed.

## 2. Materials and methods

### 2.1. Coating fabrication

TiO_2_ and SrO nanopowders were used as starting materials. 10% SrO–TiO_2_, 20% SrO–TiO_2_ and 30% SrO–TiO_2_ composite powders were produced by mixing using planetary ball mill. Coatings were deposited on biomedical grade Ti alloy (Baoji Junhang Metal Material Co., Ltd. Shanxi, China) with a diameter of 15 mm and 1 mm thickness by an atmospheric plasma spraying system (9M, Sulzer Metco, USA). Before plasma spraying, the Ti alloy substrates were ultrasonically cleaned in absolute ethanol and sandblasted with brown corundum. For cell culture experiments, the Ti alloy discs without any coatings and with pure TiO_2_ coating were used as controls. The main parameters used in this study to prepare the TiO_2_, 10% SrO–TiO_2_, 20% SrO–TiO_2_ and 30% SrO–TiO_2_ coatings are listed as follows: spraying power was 42 kW, Ar flow rate was 40 L min^−1^, H_2_ flow rate was 12 L min^−1^, spraying distance was 100 mm and powder feed rate was 30 g min^−1^.

### 2.2. Coating characterization

#### 2.2.1. X-ray diffraction (XRD) and scanning electron microscopy (SEM)

The phase structures of the coatings were conducted by X-ray diffraction (XRD, D/max 2500PC, Rigaku, Japan) with Cu Kα radiation (*λ* = 1.5418 Å) in the range of 20–80° (2*θ*). Scanning electron microscopy (SEM, S-3400, Japan) was used to examine the surface morphology.

#### 2.2.2. Ion release profile

The coatings were immersed in 1 mL of α-minimum essential medium (α-MEM, Hyclone, USA) (pH = 7.4) at 37 °C. The medium was refreshed every 3 d. At each time point (3, 6 and 9 days), the culture medium was collected for measurement. The ion concentration of Ti and Sr in the culture medium was measured by inductively coupled plasma atomic emission spectroscopy (ICP-OES).

#### 2.2.3. *In vitro* cell-free mineralization

The as-sprayed TiO_2_ and the SrO doped TiO_2_ coatings were cleaned in an ultrasonic bath with ethanol and distilled water. Modified simulated body fluid (2× SBF) with Ca and P ion concentrations double those in the normal simulated body fluid (SBF). The samples were soaked in the 2× SBF at 37 °C for 14 d without stirring and the solution were refreshed every 7 days. The 2× SBF solution containing 142.0 mmol L^−1^ Na^+^, 5.0 mmol L^−1^ K^+^, 1.5 mmol L^−1^ Mg^2+^, 5.0 mmol L^−1^ Ca^2+^, 148.5 mmol L^−1^ Cl^−^, 4.2 mmol L^−1^ HCO_3_^−^ and 2.0 mmol L^−1^ HPO_4_^2−^ and 0.5 mmol L^−1^ SO_4_^2−^ was prepared by dissolving the reagents of NaCl, NaHCO_3_, KCl, K_2_HPO_4_·H_2_O, MgCl_2_·6H_2_O, CaCl_2_ and Na_2_SO_4_ into distilled water. The 2× SBF solution was buffered at pH 7.4 with Tris and HCl.^23^

#### 2.2.4. Electrochemical measurements

The corrosion resistance of the coatings were measured by the Autolab electrochemical workstation (PGSTAT 302N, METROHM, Swiss) in SBF solution using a three-electrode configuration comprising an Ag/AgCl electrode as the reference electrode, a platinum rod as the counter electrode, and the sample as the working electrode. The measurements were performed at room temperature with a scanning rate of 5 mV s^−1^. The corrosion rate was calculated based on the following equation.^24^1
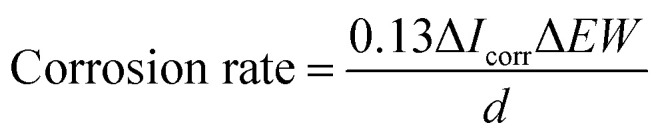


### 2.3. Biological test

#### 2.3.1. Cell culture and seeding

Rats bone marrow mesenchymal stem cells (rBMSCS) was isolated from SD rat bone marrow. SD rats were purchased from Guangdong Medical Laboratory Animal Center (Guangzhou, China). Briefly, bone marrow was rinsed out with complete culture medium consisting of α-MEM, 10% fetal bovine serum (FBS, Gibico, USA) and 1% antibiotics (100 μg mL^−1^ gentamycin and 100 U mL^−1^ penicillin). When reaching 80–90% confluence, cells were digested by 0.25% trypsin, collected by centrifugation and diluted to the desired density in culture medium. 1 mL of cell suspension with a density of 2 × 10^4^ cells per cm^2^ was added onto each samples placed in 24-well cell culture plates. The medium was refreshed every two days.

#### 2.3.2. Initial cellular attachment

To evaluate the cell attachment, cells were cultured on the samples for 24 h, and then were fixed with 2.5% glutaraldehyde in 30 min. For SEM, cell were washed with PBS twice and then dehydrated in gradient ethanol solution with final drying by isoamyl acetate. For immunofluorescent staining, the fixed cells were permeabilized with 0.1% (v/v) Triton X-100 for 7 min, and the unspecific staining was blocked by incubation in 1% BSA for 30 min. For focal adhesion staining, the specimens were incubated overnight at 4 °C with 2 μg mL^−1^ of mouse anti-mouse vinculin primary antibody (Abcam, UK) diluted in 1% BSA/PBS solution. After three washes, the sample was then incubated for 1 h with 2 μg mL^−1^ of goat anti-mouse IgG H&L (Alexa Fluor® 647) (Abcam, UK). The actin cytoskeleton was labeled by Phalloidin-FITC (Sigma, USA) for 45 min, and the cell nucleus was stained by 4′,6-diamidino-2-phenylindole dihydro-chloride (DAPI, Sigma, USA) for 5 min. The stained cells were observed under the fluorescence microscope (OLYMPUS-BX53, Japan).

#### 2.3.3. Cell proliferation

Cell Viability Kit-8 (CCK-8, Beyotime, China) was used to evaluate the cell proliferation on the coated Ti alloys. After incubation for 3 and 7 days, culture medium was removed and replaced by 10% CCK-8 working solution, followed by 2 h incubation. The OD values at 450 nm were read by a multi-well plate reader (Thermo Scientific Multiskan GO, Thermo).

#### 2.3.4. Alkaline phosphates (ALP) activity assay

For alkaline phosphatase (ALP) activity evaluation, after 14 days' incubation, the medium was removed from the cell culture plates and washed with PBS twice, followed by 30 min incubation in 200 μL of 1% Triton X-100 solution containing 100 mM phenylmethanesulfonyl fluoride (PMSF). Then 50 μL of the cell lysates was transferred to a 96-well plate and incubated for 2 h at 37 °C with 200 μL of *p*-nitrophenyl phosphate substrate solution (pNPP, Sigma, USA). ALP activity was quantified according to the absorbance at a wavelength of 405 nm. The total protein content in each cell lysate was determined using a BCA protein assay kit, to which ALP activity was normalized.

#### 2.3.5. Cell differentiation

RBMSCs were seeded onto the Ti6Al4V, TiO_2_ and the SrO doped TiO_2_ coatings at density of 2 × 10^4^ cells per cm^2^ and cultured for 7 and 14 days. The total RNA isolation and qPCR were performed strictly following the instruction from the assay kit provider. The forward and reverse primers of the selected genes are listed in Table 1. Detailed information about the experimental procedure can be found in our previous work.^25^

**Table 1:** Primer sequences for polymerase chain reaction

Gene	Sequences (5′–3′)
RUNX-2	F:ATCCAGCCACCTTCACTTACACC
	R:GGGACCATTGGGAACTGATAGG
COL-I	F:CTGCCCAGAAGAATATGTATCACC
	R:GAAGCAAAGTTTCCTCCAAGACC
OPN	F:GACGGCCGAGGTGATAGCTT
	R:CATGGCTGGTCTTCCCGTTGC
OCN	F:GCCCTGACTGCATTCTGCCTCT
	R:TCACCACCTTACTGCCCTCCTG
GAPDH	F:GGCACAGTCAAGGCTGAGAATG
	R:ATGGTGGTGAAGACGCCAGTA

### 2.4. Statistical analysis

For statistical analysis, SPSS 17.0 program was used and the data were expressed as mean ± SD. Levene's test was performed to determine the homogeneity of variance for all the data. Tukey HSD *post hoc* tests were used for the data with homogeneous variance. Tamhane's *T*2 *post hoc* was employed in the case that the tested group did not have a homogeneous variance. A *p*-value of less than 0.05 was considered significant.

## 3. Results

### 3.1. XRD analysis

**Fig. 1:**
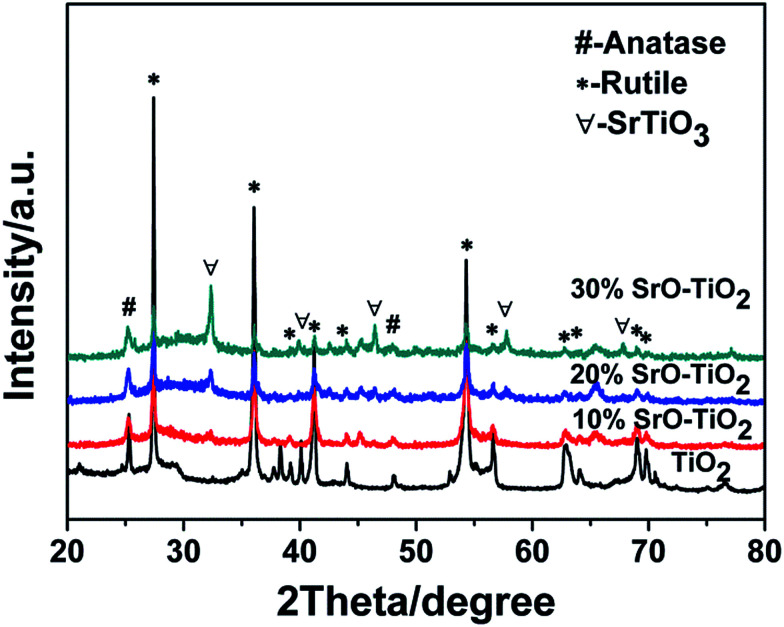
XRD patterns of the TiO_2_ and SrO–TiO_2_ coatings.

Fig. 1 shows the XRD patterns of the TiO_2_, 10% SrO–TiO_2_, 20% SrO–TiO_2_ and 30% SrO–TiO_2_ coatings on Ti alloy substrates. In the diffraction pattern of the TiO_2_ coating, peaks at 2*θ* values of 27.4°, 36.1°, 41.2°, 54.3° and 62.7° are assigned to (110), (101), (111), (211) and (002) crystalline planes of rutile TiO_2_. For the doped TiO_2_ coatings, the characteristic peaks of SrTiO_3_ at 32.4° and 39.9° were found, and their intensity increases as the amount of SrO in the coating increases. The relative ratio of SrTiO_3_/TiO_2_ in the 10% SrO–TiO_2_, 20% SrO–TiO_2_, 30% SrO–TiO_2_ coatings, calculated based on the relative peak intensity was 0.067, 0.246, 0.915, respectively. It is noted that with increase in the Sr amount in the coating, the intensity of diffraction peaks of rutile weakened, whereas those of anatase (25.3°) strengthened, indicating more anatase TiO_2_ appeared in the SrO doped TiO_2_ coatings.

### 3.2. Morphology and structural characterization

**Fig. 2:**
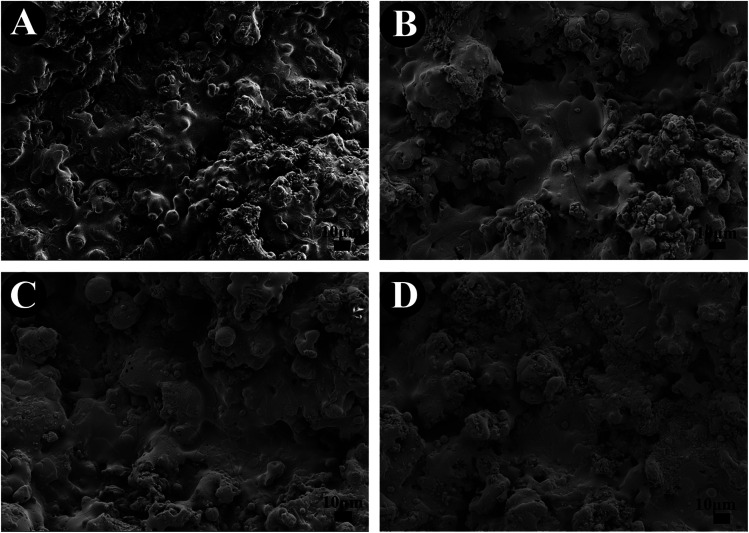
SEM images of the TiO_2_ (A), 10% SrO–TiO_2_ (B), 20% SrO–TiO_2_ (C) and 30% SrO–TiO_2_ (D) coatings.

The SEM images of the surface morphology of the TiO_2_-based coatings are showed in Fig. 2. All the coatings show a typical morphology of a plasma sprayed coating, having a rough surface with a surface roughness of 4–6 μm (Fig. 2A–D). No significant difference was found among the TiO_2_, 10% SrO–TiO_2_, 20% SrO–TiO_2_ and 30% SrO–TiO_2_ coatings.

### 3.3. Electrochemical measurements

**Fig. 3:**
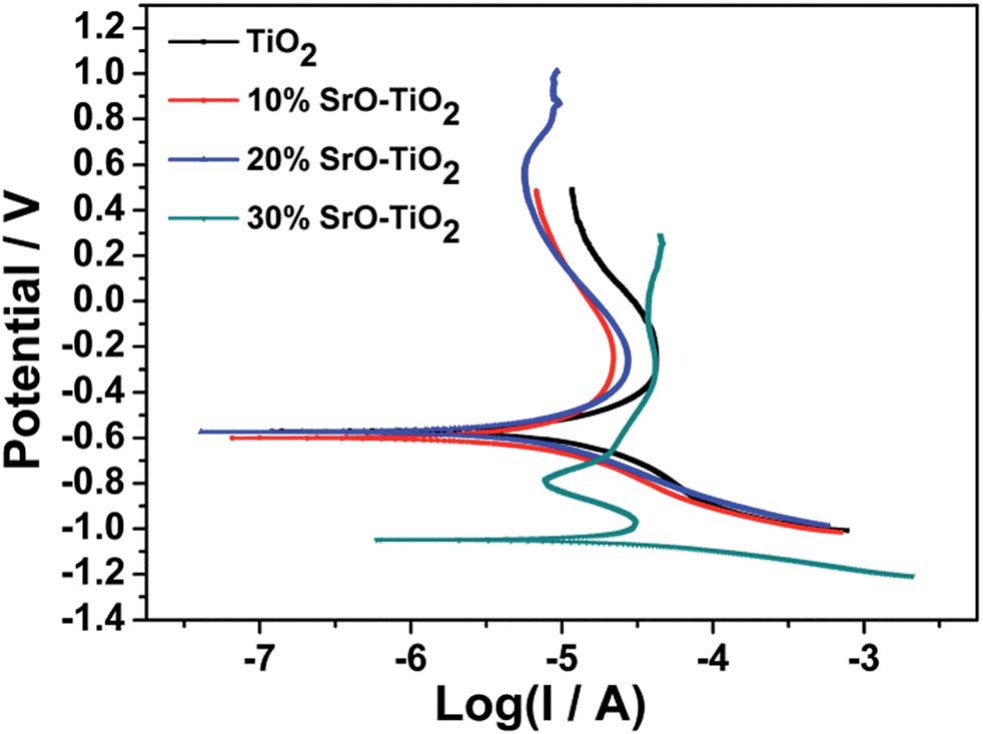
Potentiodynamic polarization curves of TiO_2_, 10% SrO–TiO_2_, 20% SrO–TiO_2_, 30% SrO–TiO_2_ coatings.

Fig. 3 displays the potentiodynamic polarization curves of the TiO_2_, 10% SrO–TiO_2_, 20% SrO–TiO_2_ and 30% SrO–TiO_2_ coatings in SBF solution. The corrosion potential (*E*_corr_), corrosion current density (*I*_corr_) and corrosion rate of the films calculated based on the polarization curves are listed in Table 2. The 20% SrO–TiO_2_ coating (−573.7 mV) and the TiO_2_ coating (−570.34 mV) have a comparable *E*_corr_, which is less negative than those of the 10% SrO–TiO_2_ (−599.7 mV) coating. The *I*_corr_ of the 20% SrO–TiO_2_ coating (1.92 μA cm^−2^) is slightly lower than that of the 10% SrO–TiO_2_ coating (2.06 μA cm^−2^), but obviously higher than that of the TiO_2_ coating (2.70 μA cm^−2^). The corrosion rate calculated based on the *I*_corr_ has the same tendency. Compared to other coatings, the 30% SrO–TiO_2_ coating has the most negative *E*_corr_ (−1048.5 mV) and the highest *I*_corr_ (7.90 μA cm^−2^)/corrosion rate. These results suggest that the 20% SrO–TiO_2_ coating has the best corrosion resistance while the 30% SrO–TiO_2_ coating has the worst. It was reported that a lower point of zero charge (PZC) of materials exhibits a higher pitting potential and better corrosion resistance.^26–29^ It was found that the PZC of SrTiO_3_ is around 8.5–9.5, higher than that of TiO_2_ (5–7),^27^ which could be one of the possible reasons for the deteriorated corrosion resistance of the 30% SrO–TiO_2_ coating. In addition, the corrosion resistance of the coating is also strongly influenced by the structural defects, such as microcracks and pores *etc.* Future work will be carried to investigate into the influence of the amount of Sr on the microstructures of the SrO–TiO_2_ coatings.

**Table 2:** *E*_corr_, *I*_corr_ and corrosion rate of the coatings

Sample	*E*_corr_ (mV)	*I*_corr_ (μA cm^−2^)	Corrosion rate (mm per year)
TiO_2_	−570.34	2.6971	0.09363
10% SrO–TiO_2_	−599.7	2.0559	0.071371
20% SrO–TiO_2_	−573.7	1.9233	0.066768
30% SrO–TiO_2_	−1048.5	7.8986	0.2742

### 3.4. *In vitro* mineralization

**Fig. 4:**
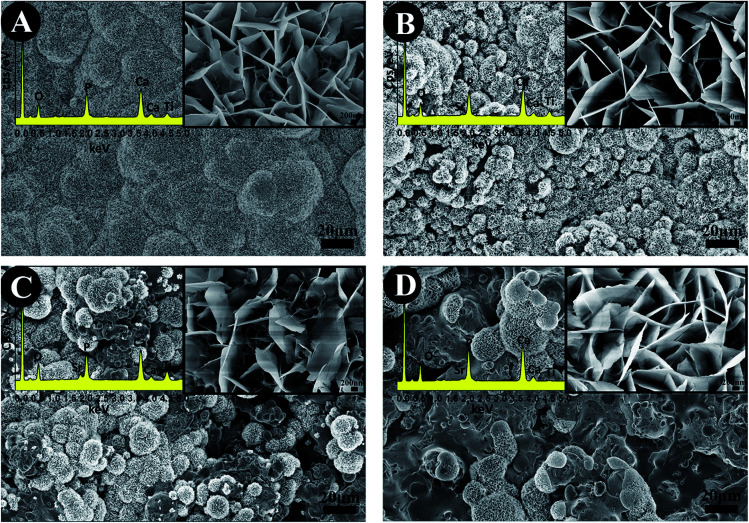
The surface morphology of the TiO_2_ (A), 10% SrO–TiO_2_ (B), 20% SrO–TiO_2_ (C), 30% SrO–TiO_2_ (D) coatings after immersion in 2× SBF for 14 days.

**Fig. 5:**
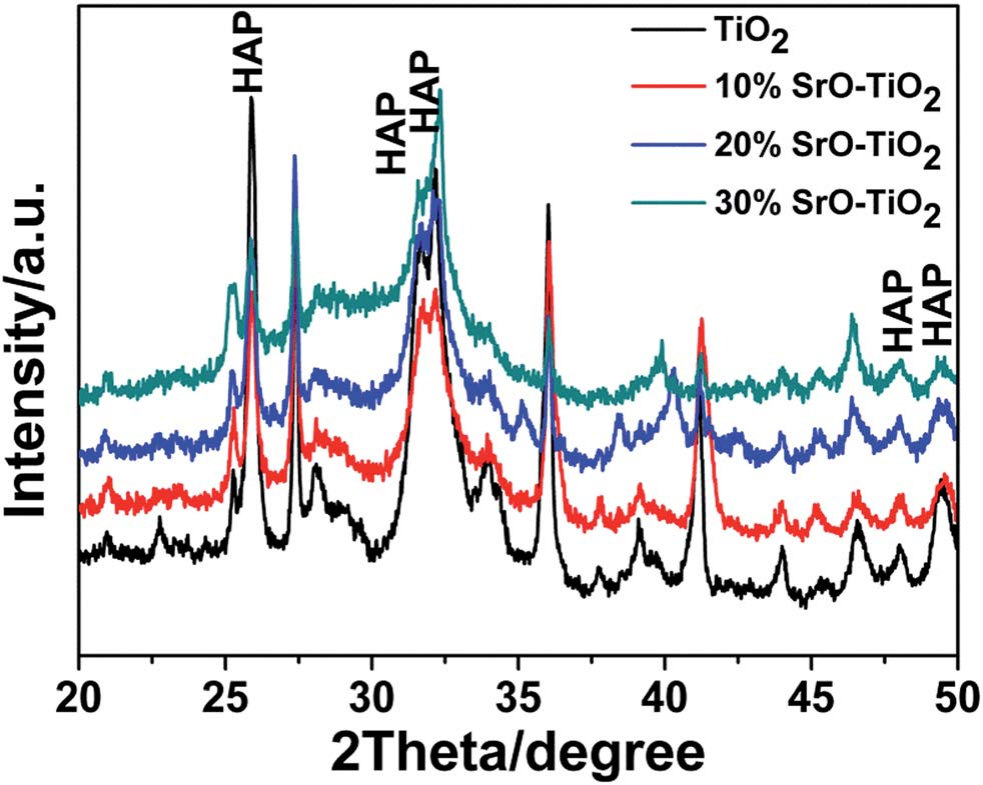
XRD patterns of the TiO_2_ coating and the SrO–TiO_2_ coatings soaked in 2× SBF for 14 days.

Fig. 4 represents the surface morphologies of the TiO_2_, 10% SrO–TiO_2_, 20% SrO–TiO_2_ and 30% SrO–TiO_2_ coatings after immersion in 2× SBF for 14 days. A large amount of granular precipitation is observed on the TiO_2_, 10% and 20% SrO–TiO_2_ coatings, while few observed on the 30% SrO–TiO_2_ coating. Under higher magnifications, it can be seen that the precipitated granules consist of nanosized plate-like crystals. EDS results prove that the precipitation is mainly composed of Ca and P (top left insets). On the TiO_2_ coating, the precipitation almost covers the whole surface, showing the best ability to induce *in vitro* mineralization. Among the SrO doped TiO_2_ coatings, the amount of precipitation formed on the 10% SrO–TiO_2_ coating seems the largest, followed by the 20% SrO–TiO_2_ coating. Fig. 5 shows XRD results of the coatings soaked in 2× SBF for 14 days. The characteristic peaks at 25.8° and 31.6° corresponding to hydroxyapatite were observed in the XRD patterns, indicating that the newly formed precipitate was hydroxyapatite. In addition, the relative intensity ratio of HAp to TiO_2_ is also found to decrease inversely with the Sr amount, consistent with what we observed from the SEM images (Fig. 4). These results suggest that the addition of SrO compromises the ability of the TiO_2_ coating to induce apatite formation. Further work will be carried to illustrate the underlying mechanisms.

### 3.5. Ion release profile in culture medium

**Fig. 6:**
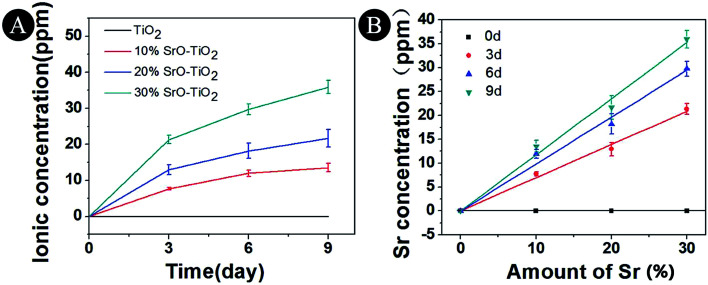
Concentrations of Sr ions released from TiO_2_, 10% SrO–TiO_2_, 20% SrO–TiO_2_, 30% SrO–TiO_2_ coatings after immersion in culture medium for 3, 6 and 9 days.

The Sr ion release behavior of the TiO_2_, 10% SrO–TiO_2_, 20% SrO–TiO_2_ and 30% SrO–TiO_2_ coatings were evaluated over 9 days and the release profile is shown in Fig. 6. It can be seen that Sr is releasable in all the doped TiO_2_ coatings, and its concentration in the immersion medium increases as the immersion time extended from 3 days to 9 days (Fig. 6A). As shown in Fig. 6B, the Sr concentration at each times for the SrO doped TiO_2_ coating is linearly related to the relative amount of SrO in the starting composite powders.

### 3.6. Cell adhesion and morphology

**Fig. 7:**
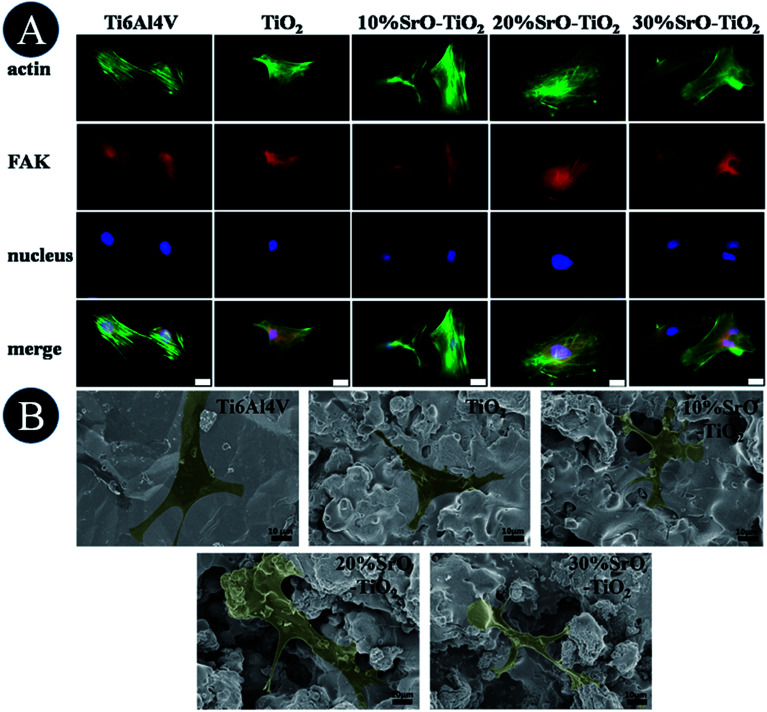
Observation of cell initial attachment on the coatings, fluorescence microscope images (A) and SEM (B) of cells cultured on Ti6Al4V, TiO_2_, 10% SrO–TiO_2_, 20% SrO–TiO_2_, 30% SrO–TiO_2_ for 24 h. Scale bar, 20 μm.

Fig. 7A shows fluorescence microscope images of the cell cultured on the Ti6Al4V, TiO_2_, 10% SrO–TiO_2_, 20% SrO–TiO_2_ and 30% SrO–TiO_2_ coatings for 24 h. Cells on all the coatings get flattened and well attach to their underlying substrates. Among these coatings, the cells cultured on the 10% SrO–TiO_2_ coating and the 20% SrO–TiO_2_ coating, especially the latter one, exhibit distinct and well-defined stress fibers and cytoskeleton.

Fig. 7B shows the SEM images of the cells cultured on the samples for 24 h. Most of the cells on the Ti, TiO_2_, 10% SrO–TiO_2_ and 20% SrO–TiO_2_ coatings exhibit a flattened polygonal shapes, while those on the 30% SrO–TiO_2_ coatings are less flattened.

### 3.7. Cell viability and proliferation

**Fig. 8:**
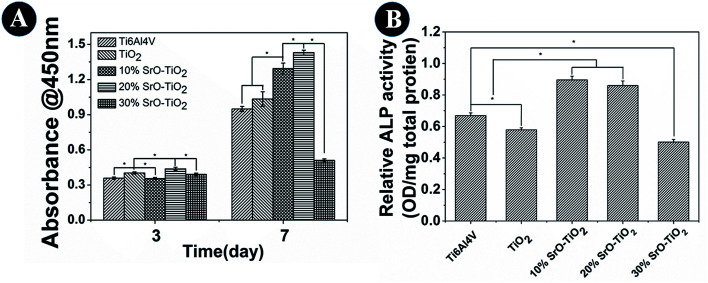
The proliferation and ALP activity and of rBMSCs cultured on the Ti6Al4V, TiO_2_, 10% SrO–TiO_2_, 20% SrO–TiO_2_, 30% SrO–TiO_2_ coatings at day 14.

Cell proliferation results are shown in Fig. 8A. At day 3, cells cultured on the 20% SrO–TiO_2_ coatings show higher proliferation rates than those cultured on the TiO_2_, 10% SrO–TiO_2_ and 30% SrO–TiO_2_ coating. After culturing for 7 days, the proliferation rate of the cells on the 20% SrO–TiO_2_ coating is still the highest, followed by that on the 10% SrO–TiO_2_ coating, while the cells on the 30% SrO–TiO_2_ coating exhibit the lowest proliferation rate. The average proliferation rate for the cells on the TiO_2_ coating is higher than that on the Ti6Al4V, but without significant statistic difference. These results indicate that Sr doping has great effects on the cell proliferation on the coatings and the 20% SrO–TiO_2_ coating has the best positive effect.

### 3.8. Alkaline phosphatase (ALP) activity

Fig. 8B shows the ALP activity of the cells cultured on the Ti6Al4V, the TiO_2_, 10% SrO–TiO_2_, 20% SrO–TiO_2_, 30% SrO–TiO_2_ coatings at day 14. It can be seen that the ALP activity of the cells cultured on the 10% SrO–TiO_2_ coating and the 20% SrO–TiO_2_ coating is comparable, which is significantly higher than those for the cells on the Ti6Al4V and the TiO_2_ coating. The ALP activity of the cells on the 30% SrO–TiO_2_ coating is lowest.

### 3.9. mRNA expression of the osteogenic-related genes

**Fig. 9:**
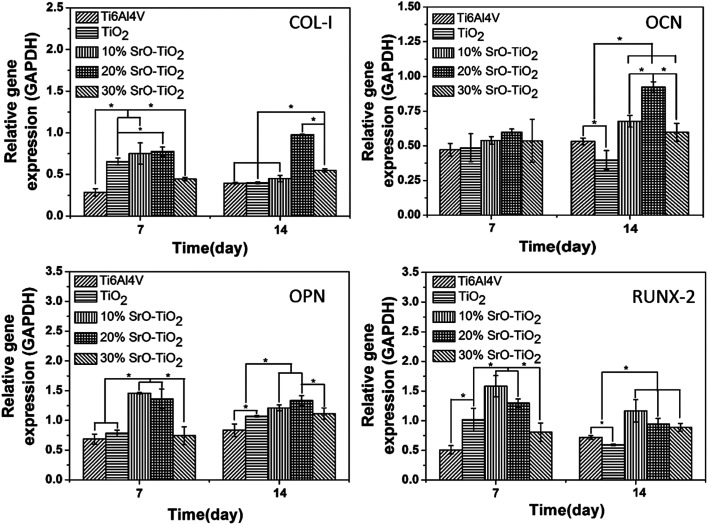
Quantitative PCR analysis of the cells cultured on TiO_2_, 10% SrO–TiO_2_, 20% SrO–TiO_2_, 30% SrO–TiO_2_ coatings for 7 and 14 days, house-keeping gene GAPDH was used as an internal control. *Statistically significant difference among different samples (*p* < 0.05).

Fig. 9 displays the expression of the osteogenic-related genes by the cells cultured on the Ti6Al4V, the TiO_2_ coating, and the SrO-doped TiO_2_ coatings. Among these genes, RUNX-2 and COL-I are early markers, while OCN and OPN are later markers for osteogenic differentiation. As shown in Fig. 9, after 7 days, the COL-I expression levels by the cells cultured on the TiO_2_ coating, the 10% SrO–TiO_2_ coating and the 20% SrO–TiO_2_ coating are significantly higher than those on the Ti6Al4V and the 30% SrO–TiO_2_ coating. At day 14, the COL-I expression level on the 20% SrO–TiO_2_ coating become the highest, which nearly doubles those for the other coatings. For OCN, no difference is found for all the samples at day 7. However, the cells cultured on the 20% SrO–TiO_2_ coating display the highest OCN expression level at day 14 and no significant difference can be seen between the 10% SrO–TiO_2_ coating and the 30% SrO–TiO_2_ coating. For OPN, its levels expressed by the cells on the 10% SrO–TiO_2_ coating and the 20% SrO–TiO_2_ coating are comparable and significantly higher than those for the other coatings at day 7. After culturing for 14 days, no significant difference can be found anymore between the 10% SrO–TiO_2_ coating and 20% SrO–TiO_2_ coating, but their expression levels are higher than the control groups. For RUNX-2, at both day 7 and 14, its expression levels by the cells cultured on the 10% SrO–TiO_2_ coating and the 20% SrO–TiO_2_ coating are comparable and higher than those by the cells on the control groups. Again, the cells cultured on the 30% SrO–TiO_2_ coating show the lowest expression level. In general, it can be concluded from these results that the 20% SrO–TiO_2_ coating are superior to the others in promoting osteogenic differentiation, while the 30% SrO–TiO_2_ coating showed obviously less osteogenic activity compared to the other doped coatings.

## 4. Discussion

In this study, we developed SrO–TiO_2_ coatings to enhance the osseointegration of the metallic orthopaedic implant. Compared to the commonly used biodegradable Sr-containing biomedical coating,^30–34^ the combination of bioactive Sr and non-degradable TiO_2_ in this study endowed the coating with an ability to release bioactive Sr (Fig. 6) and thus obviously improving the osteogenic activity (Fig. 9). It should be noted that the release of Sr is not combined with the release of Ti, indicating that the release of Sr in the developed coating system is selective, which will benefit the long-term stability of the implant.

**Fig. 10:**
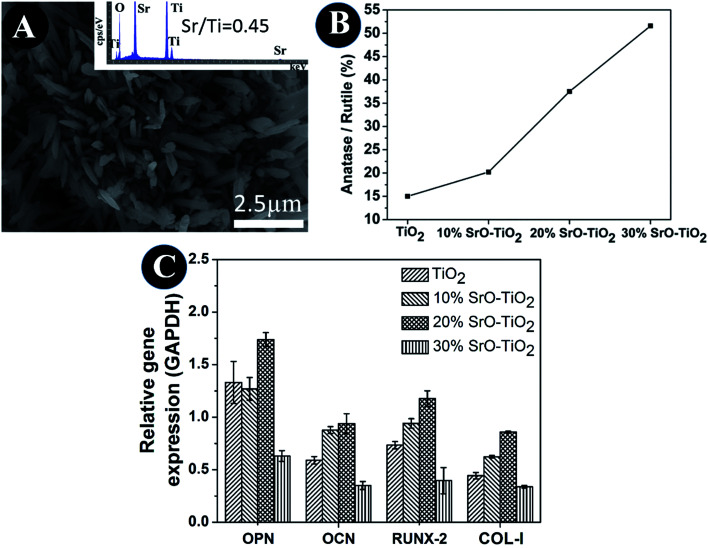
SEM and EDS of crystals precipitated on its surface after incubation with cells for 24 h (A), the ratio of anatase and rutile phase in the composite coatings (B), quantitative PCR analysis of the cells cultured on TiO_2_, 10% SrO–TiO_2_, 20% SrO–TiO_2_, 30% SrO–TiO_2_ coatings dissolution products (C).

In the SrO-doped TiO_2_ coatings, the Sr exists in two different configurations: Sr intercalated in TiO_2_ lattice and SrTiO_3_. In the 10% SrO–TiO_2_ coating, the amount of SrTiO_3_ calculated from XRD patterns is around 6.31 wt%. The atomic ratio of the Sr in the SrTiO_3_ relative to the total amount of Ti in this coating is 0.017, much less than the theoretic value of 0.052, suggesting that there must be some part of Sr (0.035) existing in other configurations. Based on the solid solution and doping theory, the most possible configuration of Sr is as interstitial atoms of the TiO_2_ lattice in SrO–TiO_2_ solid solution (Fig. 1). When the SrO amount increases to 20%, the percent of SrTiO_3_ is around 19.7 wt%, leading to a Sr/Ti of 0.061, which is nearly half of the theoretic value (0.115). Therefore, there are also a large part of Sr existing as an interstitial solute in the SrO–TiO_2_ solid solution.^35,36^ However, in the 30% SrO–TiO_2_ coating, the ratio of Sr in SrTiO_3_ relative to the total Ti in the coating (0.193) is very close to the theoretic value (0.198), therefore, it can been concluded that Sr in this coating exists only in the form of SrTiO_3_. Therefore, the ion release of Sr from the SrO-doped TiO_2_ coating is contributed by two different Sr configurations. For the 10% SrO–TiO_2_ coating and the 20% SrO–TiO_2_ coating, the released Sr is from both SrTiO_3_ and the Ti_*y*_Sr_2−2*y*_O_2_ solid solution, whereas that for the 30% SrO–TiO_2_ coating is solely from SrTiO_3_. As shown in Fig. 6, the linear release behavior was achieved for all the coatings by the two Sr configurations, suggesting that the Sr release could be precisely controlled by adjusting the amount of Sr and the Sr configurations in the coating. However, it should be noted that there are numerous nanosized crystals precipitated on the surface of the 30% SrO–TiO_2_ coating after incubation with cells for 24 h, as shown in Fig. 10A. Based on the EDS results, the Sr/Ti ratio of the newly formed crystals is around 0.45, nearly doubles the theoretical value of 0.19, implying that the newly formed crystals is a Sr-rich compound. Based on this, it can be deduced that the Sr released from the 30% SrO–TiO_2_ coating possibly led to an increased supersaturation degree with respective to a certain of Sr-containing compound, which “recycles” the released Sr ions, and ultimately decreased the ion concentration in their extracts.^37^ Therefore, the linear release behavior for the 30% SrO–TiO_2_ with respective to the SrO amount is also contributed by the newly formed layer of the crystals.

The TiO_2_ powders we used in this study are composed of 85% anatase and 15% rutile TiO_2_. However, it can be seen that the relative amount of the anatase TiO_2_ was significantly reduced after plasma spaying, indicating that plasma spraying promotes the phase transformation from anatase to rutile (Fig. 10B). With the increase in the amount of Sr in the coating, the relative amount of the anatase TiO_2_ increases, applying that the Sr incorporation can suppress the anatase to rutile transformation. It is well-known that rutile and anatase are two most common phases for TiO_2_.^38^ V. Sollazzo reported that anatase TiO_2_ has better bioactivity compared to the rutile phase.^39^ Although no direct evidence is obtained in this study to proof the contribution of the anatase TiO_2_ on the bioactivity, the rutile to anatase transformation caused by Sr doping still could be a merit of our coating design, which might provide some guidance for further biomedical coating design based on the TiO_2_ material. Based on the biological results, we found that the 20% SrO–TiO_2_ coating not only promoted the proliferation of rBMSCs (Fig. 8A), but also enhanced their osteogenic differentiation, as indicated in the ALP activity (Fig. 8B) and gene expression levels (Fig. 9), which is closely related to the Sr ions released from the coating. To illustrate the dominant effects of the Sr ion on the enhancement of the osteogenic activity, we used the extract of the samples to culture the rBMSCs to evaluate the biological effects of the dissolution products. The expression levels of bone-related genes (OPN, RUNX-2, COL-I and OCN) by the cells cultured for 7 days in the extracts were presented in Fig. 10C. It can be seen that the cells cultured by the extract of the 20% SrO–TiO_2_ coating express the highest levels of these genes, whereas those cultured in the extract of the 30% SrO–TiO_2_ coating express the lowest levels. The consistency of the gene expression results for the cells directly cultured on the coating surface and incubated with the coating extracts further validates the importance of Sr ions for the osteogenic activity. Similar to the biological drugs, the effects of the bioactive ions are also does-dependent.^10,40,41^ Therefore, based on the biological results, we may conclude that the Sr ions released from the 20% SrO–TiO_2_ coating is the optimal and the Sr ions from the 30% SrO–TiO_2_ coating are possibly over-dosed.

## 5. Conclusion

In summary, non-degradable bioactive coating was designed based a SrO–TiO_2_ system in this work, which is able to selectively release Sr ions. It was proven that the release of Sr ions is dependent on the Sr configurations in the coating, which has great influence on the osteogenic activity of the bone cells. Sr mainly exists as interstitial atoms in a solid solution of Ti_*y*_Sr_2−2*y*_O_2_ when its amount is less than 10 wt%, and only SrTiO_3_ configuration appears when the Sr amount is higher than 30%. Both configurations present in the coatings with a Sr amount lying in between. We found that the addition of Sr compromises the *in vitro* mineralization ability of the TiO_2_ coating, inversely proportional to its amount. However, its beneficial effects on osteogenesis enhancement are prominent. It was proved that the 20% SrO–TiO_2_ coating shows the best capacity of enhancing cellular proliferation and osteogenic differentiation, pointing out its potential application as an orthopaedic implant coating.

## Conflicts of interest

There are no conflicts to declare.

## Supplementary Material
